# N^6^-methyladenosine–modified RNA acts as a molecular glue that drives liquid–liquid phase separation in plants

**DOI:** 10.1080/15592324.2022.2079308

**Published:** 2022-05-27

**Authors:** Hong Gil Lee, Jiwoo Kim, Pil Joon Seo

**Affiliations:** a Department of Chemistry, Seoul National University, Seoul, Korea; b Plant Genomics and Breeding Institute, Seoul National University, Seoul, Korea; c Research Institute of Basic Sciences, Seoul National University, Seoul, Korea

**Keywords:** Liquid-liquid phase separation, m6A modification, m6A reader, ECT

## Abstract

Liquid-like condensates are organized by multivalent intrinsically disordered proteins and RNA molecules. We here demonstrate that N^6^-methyladenosine (m^6^A)-modified RNA is widespread in establishing diverse plant cell condensates. Several m^6^A-reader proteins contain putative prion-like domains, and the *ect2/3/4* mutant exhibited reduced formation of key nuclear and cytoplasmic condensates in *Arabidopsis*.

Liquid–liquid phase separation (LLPS) facilitates the formation of condensed membraneless intracellular compartments. These liquid-like condensates form local biochemical reaction centers with spatiotemporal specificity and dynamic, reversible behaviors. They usually function as cellular reaction centers for efficiently organizing stimulus-responsive biological processes.^1^ LLPS requires interactions among multivalent molecules. For example, multivalent proteins containing multiple modular interaction domains and/or disordered regions are necessary for LLPS.^2^ Specifically, intrinsically disordered proteins (IDPs), which usually contain intrinsically disordered regions (IDRs), low-complexity sequence domains (LCDs), or prion-like domains (PrDs), drive LLPS.^1^ Also, multivalent nucleic acids, which contain multiple nucleic acid–binding and protein-binding regions, participate in LLPS.^1,3^ In particular, RNA molecules greatly alter the biophysical properties of liquid droplets and are unequivocally involved in vast repertoires of LLPS-dependent condensate formation.^1^ Accordingly, RNA-binding proteins (RBPs) are rich in condensates and contribute to the organization of cellular condensates.^1,3^

The N^6^-methyladenosine (m^6^A) modification of mRNAs increases LLPS in mammalian cells, which facilitates condensate formation to control transcription, RNA processing, and translation.^4^ The LLPS mediated by m^6^A-modified RNAs depends on YTHDF m^6^A-reader proteins that each contains both an m^6^A-recognition domain (YTH domain) at the C-terminus and an IDR domain at the N-terminus.^5^ Therefore, polymethylated mRNAs act as multivalent scaffolds that bind YTHDF proteins with IDRs, leading to LLPS in mammalian cells.^4^

Because LLPS occurs in nearly all living organisms, m^6^A modification is probably also crucial for condensate formation in plant cells. A recent study by Xu et al. made an important step forward in plant LLPS, which shows a potential contribution of m^6^A modification of RNAs to liquid-like condensate formation.^6^ The FLOWERING CONTROL LOCUS A (FCA) condensate, a collection of RBPs and 3′-RNA processing machineries, promotes proximal polyadenylation of multiple nuclear RNAs, including *COOLAIR*.^3^ It is noteworthy that the FCA protein interacts with m^6^A writers (such as MTA and FIP37)^3^ and that m^6^A writers in the FCA body may deposit m^6^A onto some target transcripts and increase LLPS of the FCA condensate.^6^ The size and number of FCA condensates are significantly reduced in the *mta* mutant, which also has reduced 3′-end RNA processing activity.^6^

It is currently unknown whether m^6^A-modified RNA drives the formation of a wide range of cellular condensates in plant cells and whether condensate formation depends on m^6^A-reader proteins. Thus, we first investigated whether m^6^A-related proteins (m^6^A readers, m^6^A writers, and m^6^A erasers) have prion-like domains, which are frequently associated with the propensity of proteins to phase separate. The *Arabidopsis* genome encodes five putative m^6^A writers, including MTA (homolog of human METTL3), MTB (METTL14), FIP37 (WTAP), VIRILIZER, and HAKAI.^7^ Moreover, putative 13 m^6^A readers^8^ and potential 13 m^6^A erasers containing ALKB homolog (ALKBH) domains have also been identified in *Arabidopsis*,^5,7^ although only some of which have been empirically proven to have such m^6^A-related functions to date.^7–12^ Notably, PLAAC analysis (Prion-Like Amino Acid Composition; http://plaac.wi.mit.edu/)^13^ revealed that while few members of m^6^A writers and erasers were predicted to have PrDs (Supplemental Figure 1, Supplemental Figure 2), a majority of m^6^A reader proteins [EVOLUTIONARILY CONSERVED C-TERMINAL REGION2-8 (ECT2-8) and ECT10] have putative N-terminal PrDs (Figure 1a and Supplemental Figure 3), besides C-terminal YTH domains. Given that PrD-containing proteins frequently undergo LLPS,^1,2^ it is plausible that m^6^A-modified RNAs can drive LLPS predominantly by recruiting PrD-containing m^6^A-reader proteins.

**Figure 1.**
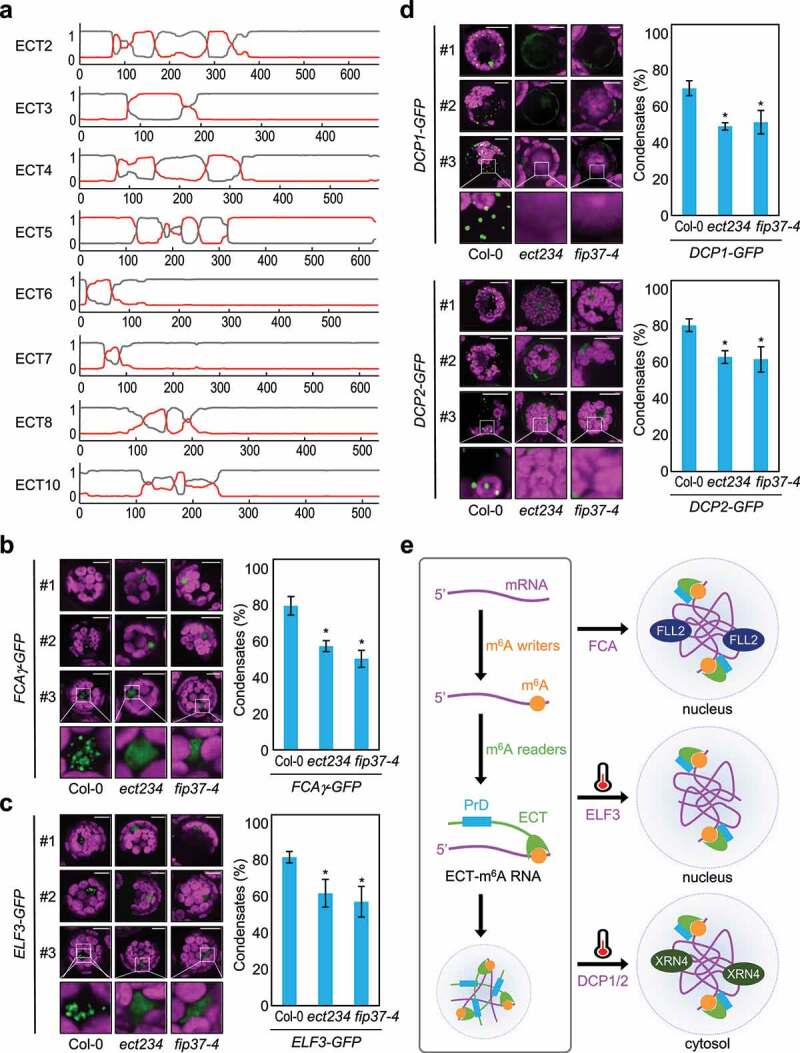
Multivalent N^6^-methyladenosine (m^6^A)–modified RNAs promote phase separation via prion-like domain (PrD)-containing ECT proteins. A) Prediction of PrDs in ECT proteins. PrDs and disordered regions in m^6^A readers were predicted by using PLAAC software. The PLAAC algorithm identifies PrD candidates by compositional similarity to domains with known prion activity. Minimum length for prion domains (L core) was set at 60 and parameter α set at 50. For background frequencies, *Arabidopsis thaliana* proteome was selected. Grey lines indicate background and red lines indicate putative PrDs. (B) ECT-dependent formation of the FCA body. (C) ECT-dependent formation of the ELF3 body. Protoplasts transfected with the *35S:ELF3-GFP* construct were incubated at 23 C for 16 h, treated at 37 °C for 2 h, and analyzed by confocal microscopy. (D) ECT-dependent formation of the processing body. Protoplasts transfected with the *35S:DCP1-GFP* and *35S:DCP2-GFP* constructs were incubated at 23 C for 16 h, treated at 39 °C for 2 h, and analyzed by confocal microscopy. In (B) to (D), mesophyll protoplasts were isolated from leaves of 2-week-old wild-type, *fip37-4 LEC1:FIP37 (fip37-4)*, and *ect234*-mutant seedlings grown under long-day conditions. Isolated mesophyll protoplasts were transiently transfected by using polyethylene glycol with the GFP-fusion constructs and then incubated in darkness for 16 h. Three biologically independent experiments were conducted. The percentages of protoplasts with condensates were quantified using total cell images (n > 40 protoplasts for each genotype in each biological replicate) obtained from three biological replicates. Among total images, representative images are shown. The statistical significance was determined using Student *t*-test (*P < 0.05). GFP signal is shown in green, and the magenta in the microscopy images is the chlorophyll autofluorescence. Scale bar = 10 μm. (E) Model of LLPS driven by m^6^A-modifed RNA. The m^6^A modification is recognized by m^6^A-binding ECT proteins with PrDs in plant cells. Because of the disordered nature of ECT proteins, m^6^A RNA–ECT protein complexes drive LLPS and enable various liquid-like condensates to form in *Arabidopsis*.

We hypothesized that PrD-containing ECT proteins contribute to biomolecular condensation in plants. The FCA body is the most well-known liquid-like condensate in plant cells and promotes proximal polyadenylation at specific poly-A sites, along with FLX-LIKE 2 (FLL2), LUMINIDEPENDENS, FLOWERING LOCUS PA (FPA), FLOWERING LOCUS Y (FY), and 3′-RNA processing components.^1,3^ To test our hypothesis, we asked whether ECT proteins are involved in the formation of the FCA body. We employed the *ect2/3/4* triple mutant, which has many developmental defects,^14^ and performed transient expression analysis by using mesophyll protoplasts. Transient expression of the *35S:FCAγ-GFP* construct in wild-type protoplasts resulted in the formation of nuclear condensates, whereas mutations in *ect2/3/4* decreased FCA body formation, similar to the m^6^A-deposition defects observed in the *fip37-4 LEC1:FIP37* mutant seedlings (Figure 1b).

To further investigate whether m^6^A-modified mRNAs generally drive LLPS to establish cellular reaction centers in plant cells, we examined whether several other key liquid-like condensates in plant cells also depend on m^6^A-modifed RNAs and on m^6^A-reader–ECT protein complexes. For instance, PrD-dependent LLPS is critical for sensing changes in ambient temperature. The PrD of ELF3 undergoes thermoresponsive LLPS and acts as a tunable thermosensor.^15^ Whereas ELF3 is diffuse in the nucleus and forms a tripartite Evening Complex with ELF4 and LUX ARRHYTHMO (LUX) at low ambient temperatures, ELF3 forms nuclear bodies in a PrD-dependent manner at high temperatures.^1,15^ We found that m^6^A RNA modification contributes to facilitating high temperature-induced formation of ELF3 condensates, in addition to LLPS of ELF3 by itself. The ELF3 bodies significantly reduced not only in the *fip37-4 LEC1:FIP37* mutant but also in *ect2/3/4* mutants at high ambient temperature (Figure 1c). We next examined whether cytosolic condensates depend also on m^6^A modification of RNAs. Cytoplasmic processing body (P-body) formation can be enhanced by heat stress ^16^(Weber et al., 2008). The P-body components DCP1 and DCP2 were consistently localized in the cytoplasmic RNA granules under heat-stress conditions (Figure 1d). In contrast, high temperature-induced condensate formation was significantly reduced in *fip37-4 LEC1:FIP37* and in *ect2/3/4* mutants (Figure 1d). Given that molecular features implicated in the formation of LLPS, such as m^6^A-modified RNAs, m^6^A RNA–related proteins, and IDPs, are conserved across plant lineages,^1,42,44^ a similar mechanism for m^6^A-dependent LLPS is pervasively exploited to create cellular hotspots in plants.

Taken together, our results indicate that polymethylated RNAs possibly recruit m^6^A-binding ECT proteins with PrDs and that the m^6^A RNA–ECT protein complex could drive LLPS in plant cells (Figure 1e). Numerous future works are required to further convince the molecular mechanism underlying cellular condensate formation in plant cells. For example, we have to rule out the possibility that *ect* mutations may lead to indirect effects on condensate formations with changes in expression of LLPS-associated genes. Furthermore, *in vitro* analysis of LLPS of PrD-containing ECT proteins is required. Domain dissection analysis for validating biochemical functions of each domain of ECT proteins is also necessary. Nonetheless, ECT-dependent LLPS is likely pervasive in liquid-like condensate formation in plant cells because several key condensates are affected by deletions of m^6^A writer and reader genes. Given that RNA transcription and modification are dynamic,^1^ m^6^A modification may efficiently regulate stimulus-responsive condensate formation, ensuring rapid adaptation of plants to changing environments.

## Materials and methods

### Plant materials and growth condition

The *Arabidopsis thaliana* ecotype Col-0 was used as wild-type control. Plants were grown under long day conditions (LDs; 16-h light/8-h dark cycles) with cool white fluorescent (120 μmol photons m^−2^ s^−1^) at 22–23°C. The *ect2/3/4* triple mutant (CS2110133) was obtained from Arabidopsis Biological Resource Center (ABRC).

### Domain analysis

Protein sequences were downloaded from The *Arabidopsis* Information Resource (TAIR; https://www.arabidopsis.org/). Protein sequences were submitted to the Batch CD-Search Tool on the NCBI portal for domain analysis. Proteins with putative prion-like domains were identified using PLAAC software (PLAAC; http://plaac.wi.mit.edu/).

### Protoplast isolation

Two-week-old seedlings grown under the LD conditions were harvested in 20 mL 0.5 M mannitol solution (90Ф plate) and incubated for 1 h at room temperature (RT). Then, the 0.5 M mannitol solution was replaced with a 20 mL enzyme solution (2% Viscozyme L, 1% Celluclast 1.5 L, 1% Pectinex Ultra SP-L in MMC, adjusted to pH 5.8 by NaOH, sterilized through a 0.2 μm syringe filtering) and incubated in the darkness for 16 h at 22–23°C. The protoplasts were collected by centrifugation at 100 g for 7 min and washed twice with the W5 solution containing 0.1% glucose, 0.08% KCl, 0.9% NaCl, 1.84% CaCl2, and 2 mM MES (pH 5.7).

### Microscopy and image analysis

Isolated protoplasts were transfected with *35S:FCAγ-GFP, 35S:ELF3-GFP, 35S:DCP1-GFP*, or *35S:DCP2-GFP* construct and incubated for 16 h in darkness. After 16-h incubation, transfected protoplasts were subjected to microscopic analysis. The fluorescence images were taken by the Confocal Quantitative Image Cytometer CQ1 (YOKOGAWA) confocal system. The excitation wavelength was 488 nm for GFP, and 635 nm for chlorophyll autofluorescence. Fluorescence emission was detected at 505–525 nm for GFP, and 660–680 nm for chlorophyll autofluorescence. The percentage of protoplasts with or without condensates from total protoplast cells (*n* > 120) was quantified.

## Supplementary Material

Supplemental Material
