# Supplementary material for: N6-methyladenosine–modified RNA acts as a molecular glue that drives liquid–liquid phase separation in plants
Source: Plant Signal Behav. 2022 May 27;17(1):2079308. doi: 10.1080/15592324.2022.2079308 (PMC9154792; doi:10.1080/15592324.2022.2079308)
Supplement: Supplemental Material [file KPSB_A_2079308_SM6035.pdf]

Figure S1

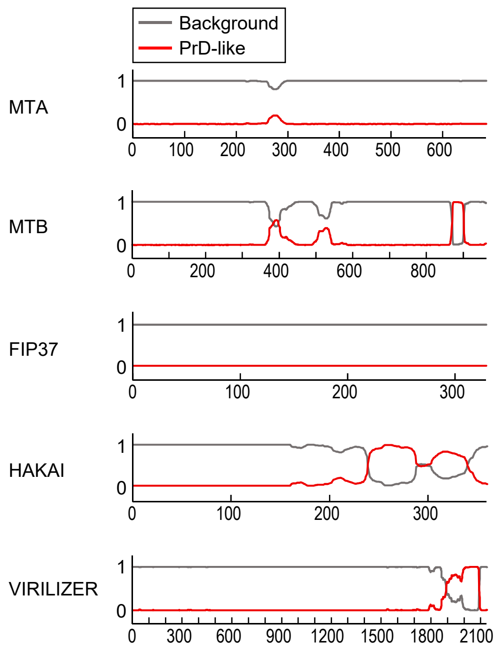

Figure S1. Prediction of PrDs in m⁶A writers. PrDs and disordered regions in m⁶A writer proteins were predicted by using PLAAC software (PLAAC; http://plaac.wi.mit.edu/). The PLAAC algorithm identifies PrD candidates by compositional similarity to domains with known prion activity.

Figure S2

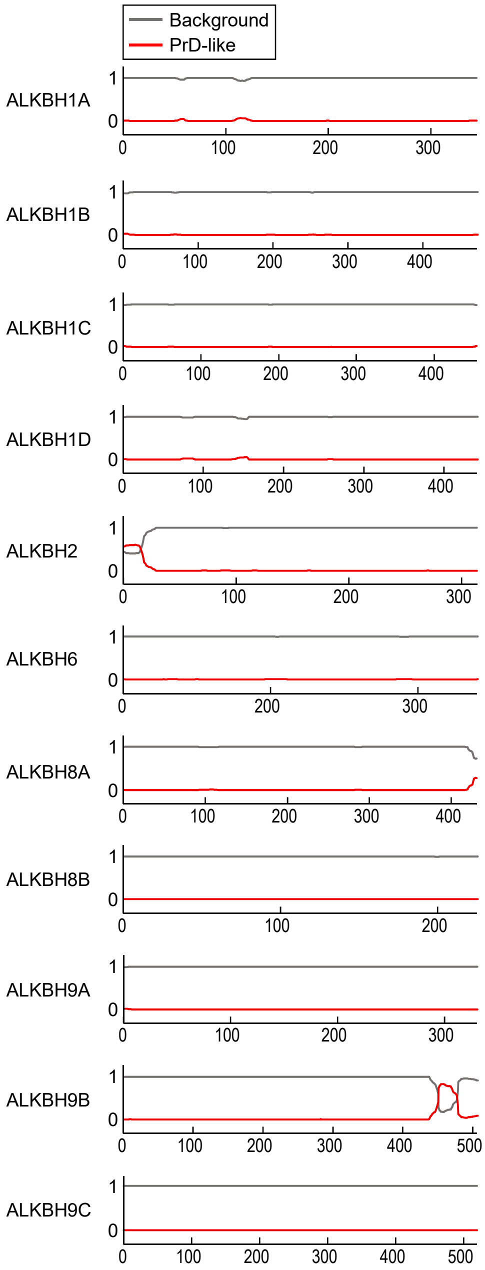

Figure S2. Prediction of PrDs in m^{6}A erasers. PrDs and disordered regions were predicted by using PLAAC software (PLAAC; http://plaac.wi.mit.edu/). The PLAAC algorithm identifies PrD candidates by compositional similarity to domains with known prion activity.

Figure S3

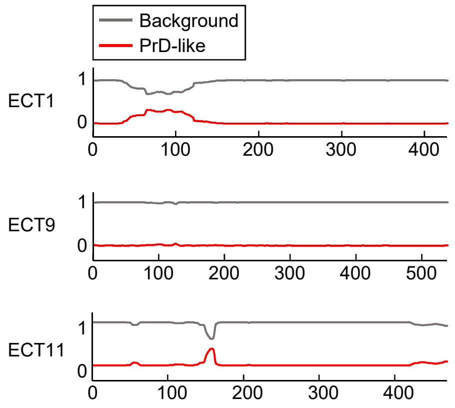

Figure S3. Prediction of PrDs in m^{6}A readers. PrDs and disordered regions were predicted by using PLAAC software (PLAAC; http://plaac.wi.mit.edu/). The PLAAC algorithm identifies PrD candidates by compositional similarity to domains with known prion activity.
